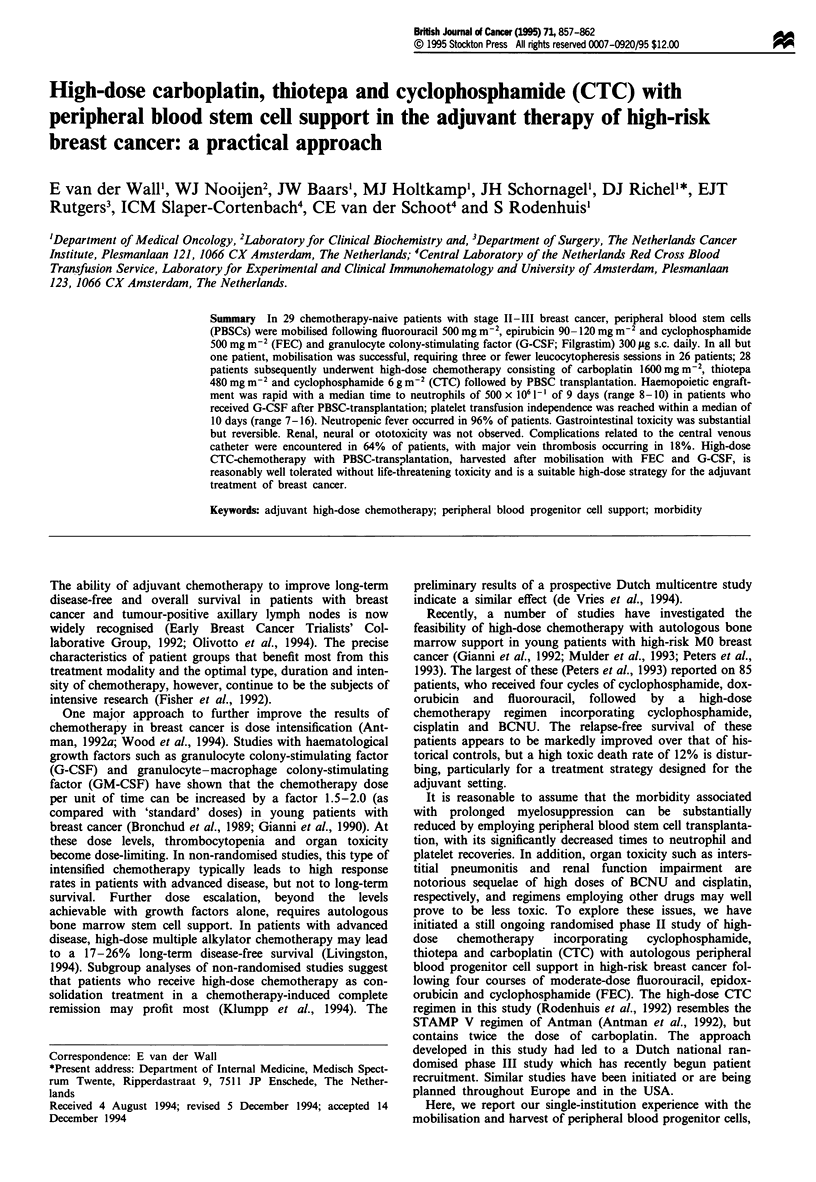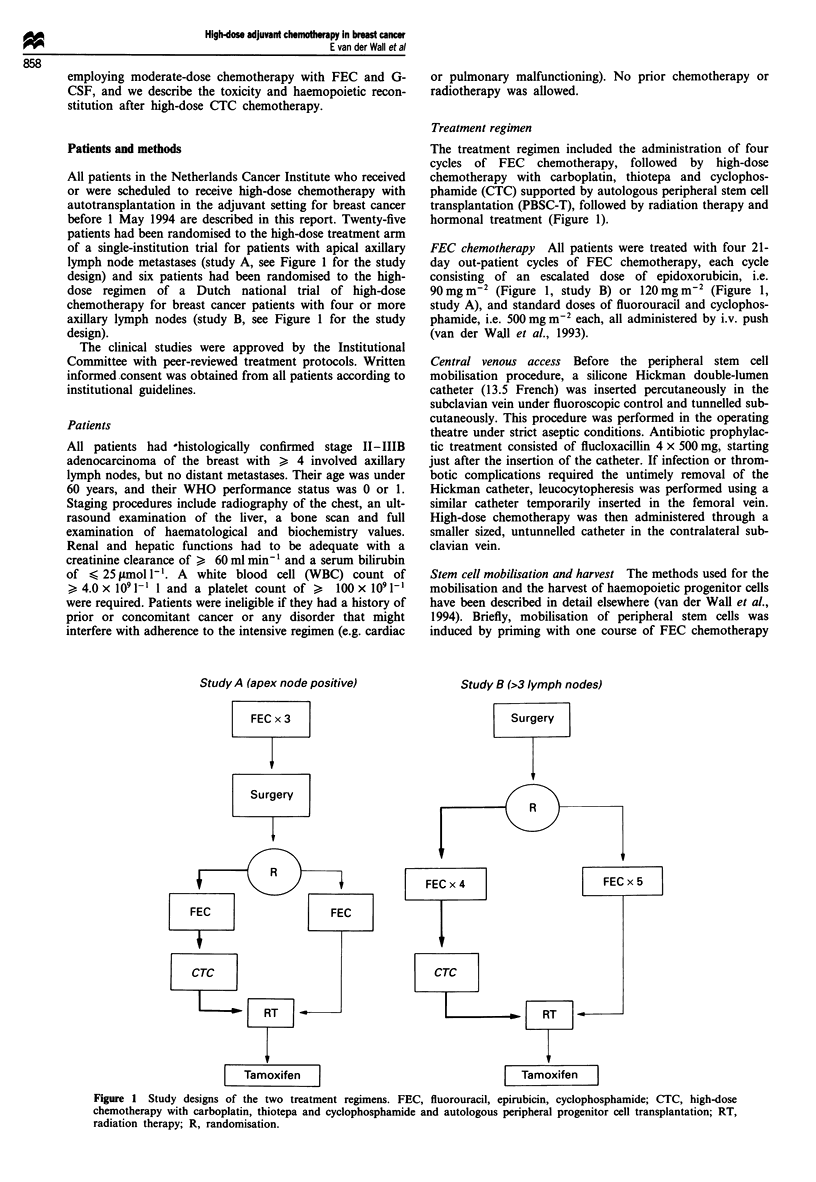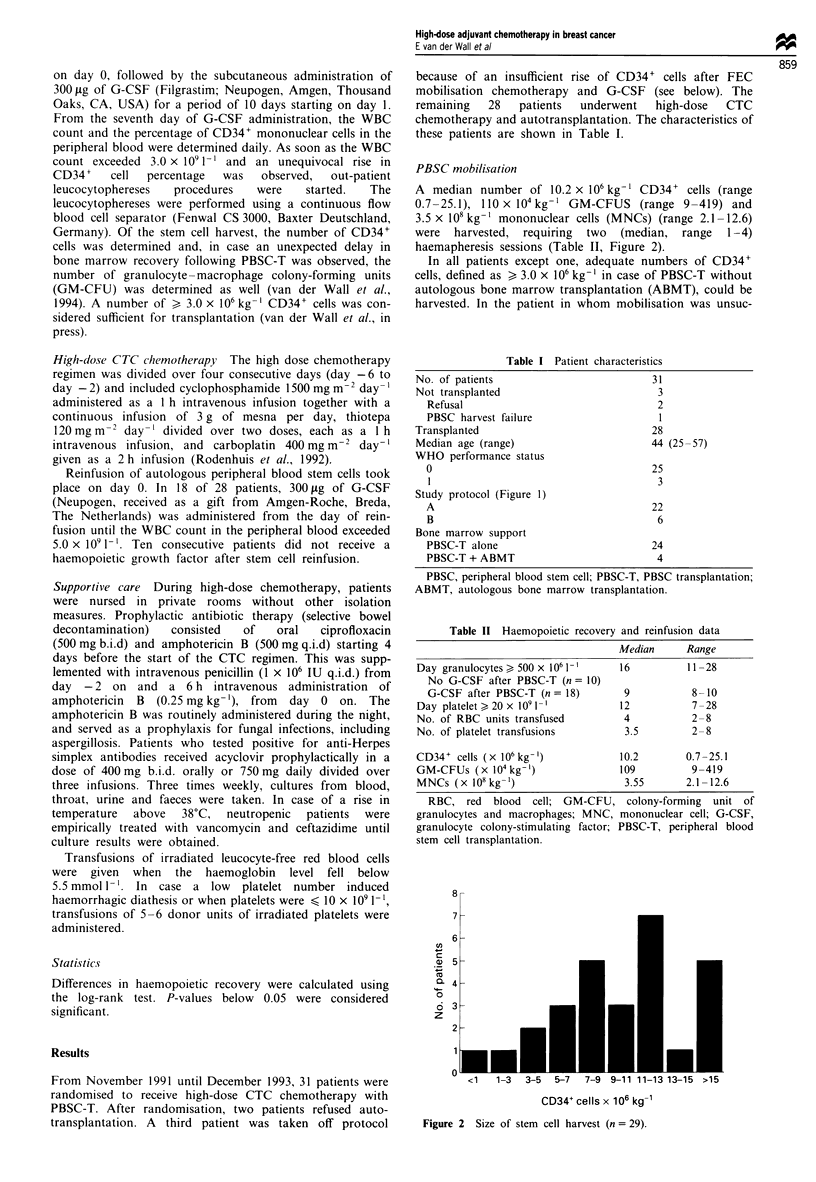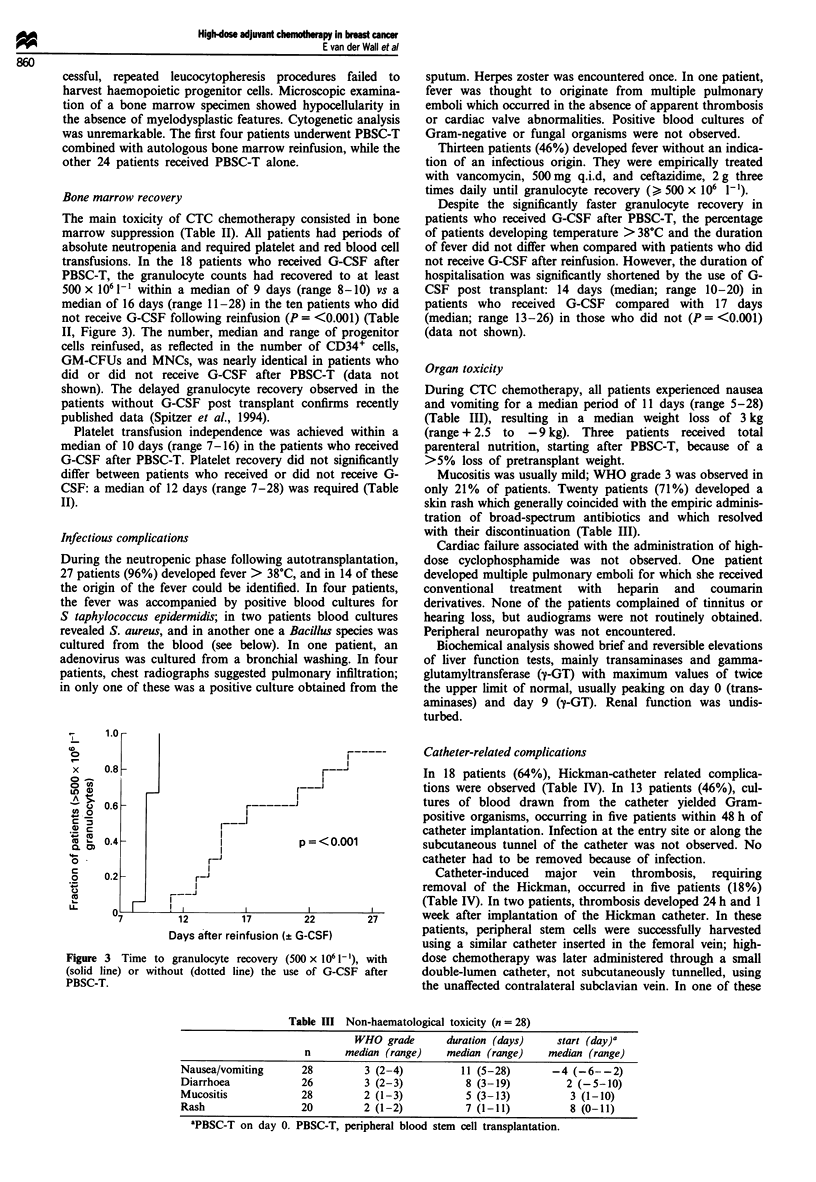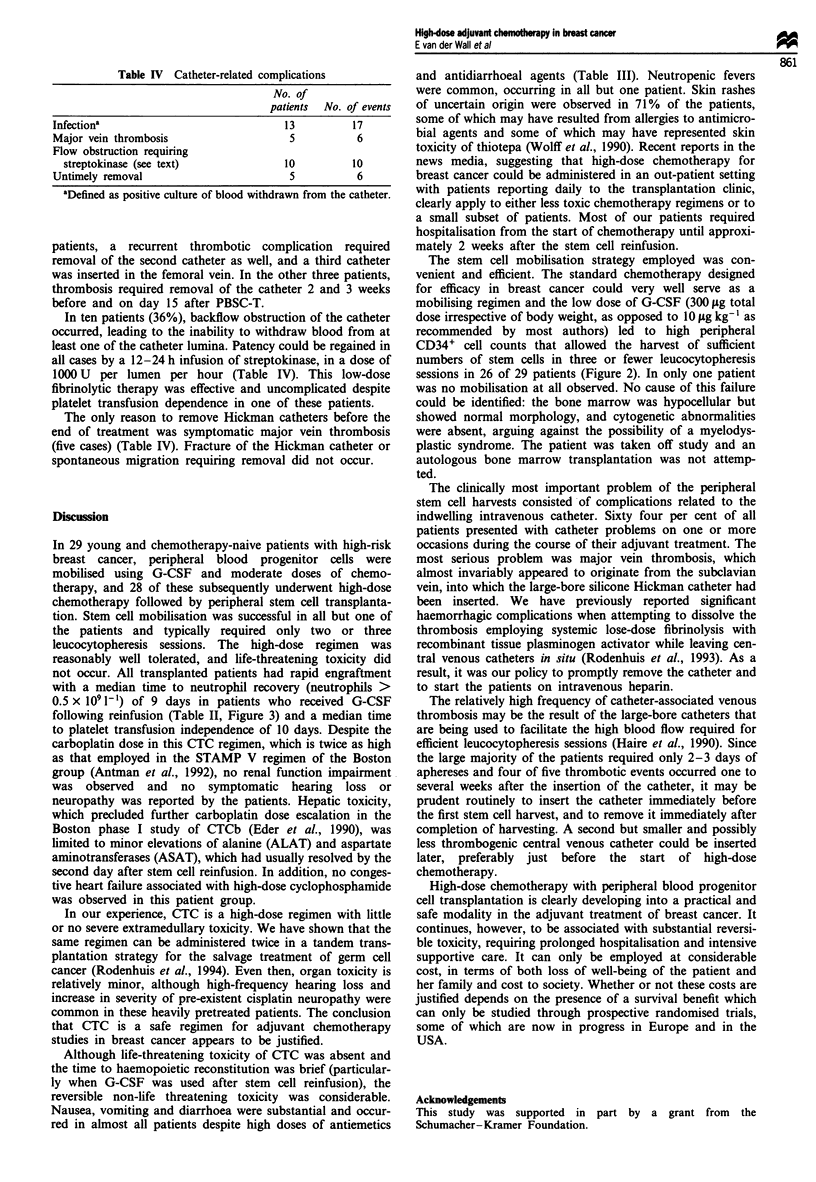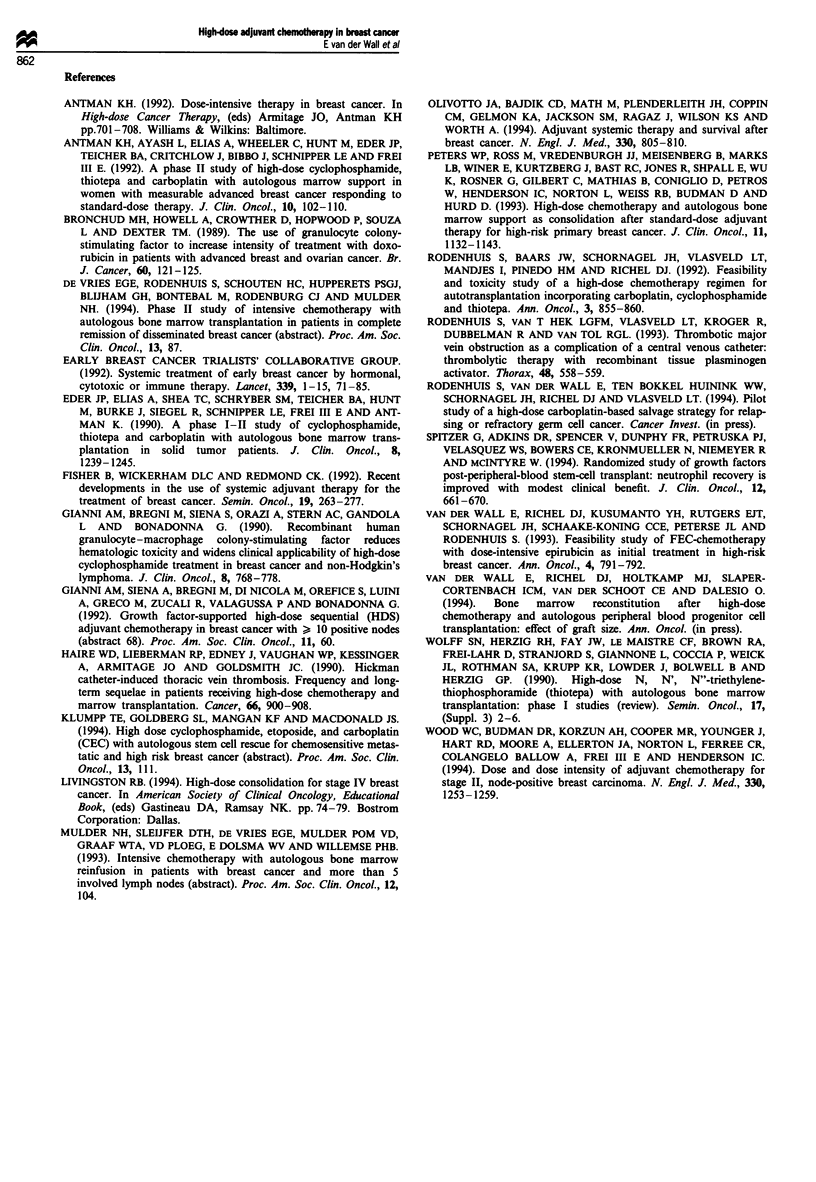# High-dose carboplatin, thiotepa and cyclophosphamide (CTC) with peripheral blood stem cell support in the adjuvant therapy of high-risk breast cancer: a practical approach.

**DOI:** 10.1038/bjc.1995.165

**Published:** 1995-04

**Authors:** E. van der Wall, W. J. Nooijen, J. W. Baars, M. J. Holtkamp, J. H. Schorangel, D. J. Richel, E. J. Rutgers, I. C. Slaper-Cortenbach, C. E. van der Schoot, S. Rodenhuis

**Affiliations:** Department of Medical Oncology, Netherlands Cancer Institute, Amsterdam.

## Abstract

In 29 chemotherapy-naive patients with stage II-III breast cancer, peripheral blood stem cells (PBSCs) were mobilised following fluorouracil 500 mg m-2, epirubicin 90-120 mg m-2 and cyclophosphamide 500 mg m-2 (FEC) and granulocyte colony-stimulating factor (G-CSF; Filgrastim) 300 microgram s.c. daily. In all but one patient, mobilisation was successful, requiring three or fewer leucocytopheresis sessions in 26 patients; 28 patients subsequently underwent high-dose chemotherapy consisting of carboplatin 1600 mg m-2, thiotepa 480 mg m-2 and cyclophosphamide 6 g m-2 (CTC) followed by PBSC transplantation. Haemopoietic engraftment was rapid with a median time to neutrophils of 500 x 10(6) l(-1) of 9 days (range 8-10) in patients who received G-CSF after PBSC-transplantation; platelet transfusion independence was reached within a median of 10 days (range 7-16). Neutropenic fever occurred in 96% of patients. Gastrointestinal toxicity was substantial but reversible. Renal, neural or ototoxicity was not observed. Complications related to the central venous catheter were encountered in 64% of patients, with major vein thrombosis occurring in 18%. High-dose CTC-chemotherapy with PBSC-transplantation, harvested after mobilisation with FEC and G-CSF, is reasonably well tolerated without life-threatening toxicity and is a suitable high-dose strategy for the adjuvant treatment of breast cancer.


British Journal of Cancer (1995) 71, 857-862
© 1995 Stockton Press All rights reserved 0007-0920/95 $12.00

### High-dose carboplatin, thiotepa and cyclophosphamide (CTC) with peripheral blood stem cell support in the adjuvant therapy of high-risk breast cancer: a practical approach

E van der Wall[1], WJ Nooijen[2], JW Baars[1], MJ Holtkamp[1], JH Schornagel[1], DJ Richel[1]*, EJT Rutgers[3], ICM Slaper-Cortenbach[4], CE van der Schoot[4] and S Rodenhuis[1]

[1]*Department of Medical Oncology,* [2]*Laboratory for Clinical Biochemistry and,* [3]*Department of Surgery, The Netherlands Cancer Institute, Plesmanlaan 121, 1066 CX Amsterdam, The Netherlands;* [4]*Central Laboratory of the Netherlands Red Cross Blood Transfusion Service, Laboratory for Experimental and Clinical Immunohematology and University of Amsterdam, Plesmanlaan 123, 1066 CX Amsterdam, The Netherlands.*

**Summary** In 29 chemotherapy-naive patients with stage II-III breast cancer, peripheral blood stem cells (PBSCs) were mobilised following fluorouracil 500 mg $m^{-2}$, epirubicin 90-120 mg $m^{-2}$ and cyclophosphamide 500 mg $m^{-2}$ (FEC) and granulocyte colony-stimulating factor (G-CSF; Filgrastim) 300 $\mu g$ s.c. daily. In all but one patient, mobilisation was successful, requiring three or fewer leucocytopheresis sessions in 26 patients; 28 patients subsequently underwent high-dose chemotherapy consisting of carboplatin 1600 mg $m^{-2}$, thiotepa 480 mg $m^{-2}$ and cyclophosphamide 6 g $m^{-2}$ (CTC) followed by PBSC transplantation. Haemopoietic engraftment was rapid with a median time to neutrophils of $500 \times 10^6 l^{-1}$ of 9 days (range 8-10) in patients who received G-CSF after PBSC-transplantation; platelet transfusion independence was reached within a median of 10 days (range 7-16). Neutropenic fever occurred in 96% of patients. Gastrointestinal toxicity was substantial but reversible. Renal, neural or ototoxicity was not observed. Complications related to the central venous catheter were encountered in 64% of patients, with major vein thrombosis occurring in 18%. High-dose CTC-chemotherapy with PBSC-transplantation, harvested after mobilisation with FEC and G-CSF, is reasonably well tolerated without life-threatening toxicity and is a suitable high-dose strategy for the adjuvant treatment of breast cancer.

**Keywords:** adjuvant high-dose chemotherapy; peripheral blood progenitor cell support; morbidity

The ability of adjuvant chemotherapy to improve long-term disease-free and overall survival in patients with breast cancer and tumour-positive axillary lymph nodes is now widely recognised (Early Breast Cancer Trialists' Collaborative Group, 1992; Olivotto *et al.*, 1994). The precise characteristics of patient groups that benefit most from this treatment modality and the optimal type, duration and intensity of chemotherapy, however, continue to be the subjects of intensive research (Fisher *et al.*, 1992).

One major approach to further improve the results of chemotherapy in breast cancer is dose intensification (Antman, 1992*a*; Wood *et al.*, 1994). Studies with haematological growth factors such as granulocyte colony-stimulating factor (G-CSF) and granulocyte-macrophage colony-stimulating factor (GM-CSF) have shown that the chemotherapy dose per unit of time can be increased by a factor 1.5-2.0 (as compared with 'standard' doses) in young patients with breast cancer (Bronchud *et al.*, 1989; Gianni *et al.*, 1990). At these dose levels, thrombocytopenia and organ toxicity become dose-limiting. In non-randomised studies, this type of intensified chemotherapy typically leads to high response rates in patients with advanced disease, but not to long-term survival. Further dose escalation, beyond the levels achievable with growth factors alone, requires autologous bone marrow stem cell support. In patients with advanced disease, high-dose multiple alkylator chemotherapy may lead to a 17-26% long-term disease-free survival (Livingston, 1994). Subgroup analyses of non-randomised studies suggest that patients who receive high-dose chemotherapy as consolidation treatment in a chemotherapy-induced complete remission may profit most (Klumpp *et al.*, 1994). The preliminary results of a prospective Dutch multicentre study indicate a similar effect (de Vries *et al.*, 1994).

Recently, a number of studies have investigated the feasibility of high-dose chemotherapy with autologous bone marrow support in young patients with high-risk M0 breast cancer (Gianni *et al.*, 1992; Mulder *et al.*, 1993; Peters *et al.*, 1993). The largest of these (Peters *et al.*, 1993) reported on 85 patients, who received four cycles of cyclophosphamide, doxorubicin and fluorouracil, followed by a high-dose chemotherapy regimen incorporating cyclophosphamide, cisplatin and BCNU. The relapse-free survival of these patients appears to be markedly improved over that of historical controls, but a high toxic death rate of 12% is disturbing, particularly for a treatment strategy designed for the adjuvant setting.

It is reasonable to assume that the morbidity associated with prolonged myelosuppression can be substantially reduced by employing peripheral blood stem cell transplantation, with its significantly decreased times to neutrophil and platelet recoveries. In addition, organ toxicity such as interstitial pneumonitis and renal function impairment are notorious sequelae of high doses of BCNU and cisplatin, respectively, and regimens employing other drugs may well prove to be less toxic. To explore these issues, we have initiated a still ongoing randomised phase II study of high-dose chemotherapy incorporating cyclophosphamide, thiotepa and carboplatin (CTC) with autologous peripheral blood progenitor cell support in high-risk breast cancer following four courses of moderate-dose fluorouracil, epidoxorubicin and cyclophosphamide (FEC). The high-dose CTC regimen in this study (Rodenhuis *et al.*, 1992) resembles the STAMP V regimen of Antman (Antman *et al.*, 1992), but contains twice the dose of carboplatin. The approach developed in this study had led to a Dutch national randomised phase III study which has recently begun patient recruitment. Similar studies have been initiated or are being planned throughout Europe and in the USA.

Here, we report our single-institution experience with the mobilisation and harvest of peripheral blood progenitor cells,

Correspondence: E van der Wall
*Present address: Department of Internal Medicine, Medisch Spectrum Twente, Ripperdastraat 9, 7511 JP Enschede, The Netherlands
Received 4 August 1994; revised 5 December 1994; accepted 14 December 1994

employing moderate-dose chemotherapy with FEC and G-CSF, and we describe the toxicity and haemopoietic reconstitution after high-dose CTC chemotherapy.

### Patients and methods

All patients in the Netherlands Cancer Institute who received or were scheduled to receive high-dose chemotherapy with autotransplantation in the adjuvant setting for breast cancer before 1 May 1994 are described in this report. Twenty-five patients had been randomised to the high-dose treatment arm of a single-institution trial for patients with apical axillary lymph node metastases (study A, see Figure 1 for the study design) and six patients had been randomised to the high-dose regimen of a Dutch national trial of high-dose chemotherapy for breast cancer patients with four or more axillary lymph nodes (study B, see Figure 1 for the study design).

The clinical studies were approved by the Institutional Committee with peer-reviewed treatment protocols. Written informed consent was obtained from all patients according to institutional guidelines.

### *Patients*

All patients had ·histologically confirmed stage II–IIIB adenocarcinoma of the breast with $\geqslant$ 4 involved axillary lymph nodes, but no distant metastases. Their age was under 60 years, and their WHO performance status was 0 or 1. Staging procedures include radiography of the chest, an ultrasound examination of the liver, a bone scan and full examination of haematological and biochemistry values. Renal and hepatic functions had to be adequate with a creatinine clearance of $\geqslant$ 60 ml min$^{-1}$ and a serum bilirubin of $\leqslant$ 25 μmol l$^{-1}$. A white blood cell (WBC) count of $\geqslant 4.0 \times 10^9$ l$^{-1}$ l and a platelet count of $\geqslant 100 \times 10^9$ l$^{-1}$ were required. Patients were ineligible if they had a history of prior or concomitant cancer or any disorder that might interfere with adherence to the intensive regimen (e.g. cardiac or pulmonary malfunctioning). No prior chemotherapy or radiotherapy was allowed.

### *Treatment regimen*

The treatment regimen included the administration of four cycles of FEC chemotherapy, followed by high-dose chemotherapy with carboplatin, thiotepa and cyclophosphamide (CTC) supported by autologous peripheral stem cell transplantation (PBSC-T), followed by radiation therapy and hormonal treatment (Figure 1).

*FEC chemotherapy* All patients were treated with four 21-day out-patient cycles of FEC chemotherapy, each cycle consisting of an escalated dose of epidoxorubicin, i.e. 90 mg m$^{-2}$ (Figure 1, study B) or 120 mg m$^{-2}$ (Figure 1, study A), and standard doses of fluorouracil and cyclophosphamide, i.e. 500 mg m$^{-2}$ each, all administered by i.v. push (van der Wall *et al.*, 1993).

*Central venous access* Before the peripheral stem cell mobilisation procedure, a silicone Hickman double-lumen catheter (13.5 French) was inserted percutaneously in the subclavian vein under fluoroscopic control and tunnelled subcutaneously. This procedure was performed in the operating theatre under strict aseptic conditions. Antibiotic prophylactic treatment consisted of flucloxacillin 4 × 500 mg, starting just after the insertion of the catheter. If infection or thrombotic complications required the untimely removal of the Hickman catheter, leucocytopheresis was performed using a similar catheter temporarily inserted in the femoral vein. High-dose chemotherapy was then administered through a smaller sized, untunnelled catheter in the contralateral subclavian vein.

*Stem cell mobilisation and harvest* The methods used for the mobilisation and the harvest of haemopoietic progenitor cells have been described in detail elsewhere (van der Wall *et al.*, 1994). Briefly, mobilisation of peripheral stem cells was induced by priming with one course of FEC chemotherapy

**Figure 1** Study designs of the two treatment regimens. FEC, fluorouracil, epirubicin, cyclophosphamide; CTC, high-dose chemotherapy with carboplatin, thiotepa and cyclophosphamide and autologous peripheral progenitor cell transplantation; RT, radiation therapy; R, randomisation.

on day 0, followed by the subcutaneous administration of 300 μg of G-CSF (Filgrastim; Neupogen, Amgen, Thousand Oaks, CA, USA) for a period of 10 days starting on day 1. From the seventh day of G-CSF administration, the WBC count and the percentage of $CD34^+$ mononuclear cells in the peripheral blood were determined daily. As soon as the WBC count exceeded $3.0 \times 10^9 l^{-1}$ and an unequivocal rise in $CD34^+$ cell percentage was observed, out-patient leucocytophereses procedures were started. The leucocytophereses were performed using a continuous flow blood cell separator (Fenwal CS 3000, Baxter Deutschland, Germany). Of the stem cell harvest, the number of $CD34^+$ cells was determined and, in case an unexpected delay in bone marrow recovery following PBSC-T was observed, the number of granulocyte–macrophage colony-forming units (GM-CFU) was determined as well (van der Wall *et al.*, 1994). A number of $\geqslant 3.0 \times 10^6 kg^{-1}$ $CD34^+$ cells was considered sufficient for transplantation (van der Wall *et al.*, in press).

*High-dose CTC chemotherapy* The high dose chemotherapy regimen was divided over four consecutive days (day −6 to day −2) and included cyclophosphamide 1500 mg $m^{-2}$ $day^{-1}$ administered as a 1 h intravenous infusion together with a continuous infusion of 3 g of mesna per day, thiotepa 120 mg $m^{-2}$ $day^{-1}$ divided over two doses, each as a 1 h intravenous infusion, and carboplatin 400 mg $m^{-2}$ $day^{-1}$ given as a 2 h infusion (Rodenhuis *et al.*, 1992).

Reinfusion of autologous peripheral blood stem cells took place on day 0. In 18 of 28 patients, 300 μg of G-CSF (Neupogen, received as a gift from Amgen-Roche, Breda, The Netherlands) was administered from the day of reinfusion until the WBC count in the peripheral blood exceeded $5.0 \times 10^9 l^{-1}$. Ten consecutive patients did not receive a haemopoietic growth factor after stem cell reinfusion.

*Supportive care* During high-dose chemotherapy, patients were nursed in private rooms without other isolation measures. Prophylactic antibiotic therapy (selective bowel decontamination) consisted of oral ciprofloxacin (500 mg b.i.d) and amphotericin B (500 mg q.i.d) starting 4 days before the start of the CTC regimen. This was supplemented with intravenous penicillin ($1 \times 10^6$ IU q.i.d.) from day −2 on and a 6 h intravenous administration of amphotericin B (0.25 mg $kg^{-1}$), from day 0 on. The amphotericin B was routinely administered during the night, and served as a prophylaxis for fungal infections, including aspergillosis. Patients who tested positive for anti-Herpes simplex antibodies received acyclovir prophylactically in a dose of 400 mg b.i.d. orally or 750 mg daily divided over three infusions. Three times weekly, cultures from blood, throat, urine and faeces were taken. In case of a rise in temperature above 38°C, neutropenic patients were empirically treated with vancomycin and ceftazidime until culture results were obtained.

Transfusions of irradiated leucocyte-free red blood cells were given when the haemoglobin level fell below 5.5 mmol $l^{-1}$. In case a low platelet number induced haemorrhagic diathesis or when platelets were $\leqslant 10 \times 10^9 l^{-1}$, transfusions of 5–6 donor units of irradiated platelets were administered.

*Statistics*

Differences in haemopoietic recovery were calculated using the log-rank test. *P*-values below 0.05 were considered significant.

### Results

From November 1991 until December 1993, 31 patients were randomised to receive high-dose CTC chemotherapy with PBSC-T. After randomisation, two patients refused autotransplantation. A third patient was taken off protocol because of an insufficient rise of $CD34^+$ cells after FEC mobilisation chemotherapy and G-CSF (see below). The remaining 28 patients underwent high-dose CTC chemotherapy and autotransplantation. The characteristics of these patients are shown in Table I.

*PBSC mobilisation*

A median number of $10.2 \times 10^6 kg^{-1}$ $CD34^+$ cells (range 0.7–25.1), $110 \times 10^4 kg^{-1}$ GM-CFUS (range 9–419) and $3.5 \times 10^8 kg^{-1}$ mononuclear cells (MNCs) (range 2.1–12.6) were harvested, requiring two (median, range 1–4) haemapheresis sessions (Table II, Figure 2).

In all patients except one, adequate numbers of $CD34^+$ cells, defined as $\geqslant 3.0 \times 10^6 kg^{-1}$ in case of PBSC-T without autologous bone marrow transplantation (ABMT), could be harvested. In the patient in whom mobilisation was unsuc-

**Table I** Patient characteristics

| | |
|---|---|
| No. of patients | 31 |
| Not transplanted | 3 |
| Refusal | 2 |
| PBSC harvest failure | 1 |
| Transplanted | 28 |
| Median age (range) | 44 (25–57) |
| WHO performance status | |
| 0 | 25 |
| 1 | 3 |
| Study protocol (Figure 1) | |
| A | 22 |
| B | 6 |
| Bone marrow support | |
| PBSC-T alone | 24 |
| PBSC-T + ABMT | 4 |

PBSC, peripheral blood stem cell; PBSC-T, PBSC transplantation; ABMT, autologous bone marrow transplantation.

**Table II** Haemopoietic recovery and reinfusion data

| | *Median* | *Range* |
|---|---|---|
| Day granulocytes $\geqslant 500 \times 10^6 l^{-1}$ | 16 | 11–28 |
| No G-CSF after PBSC-T ($n = 10$) | | |
| G-CSF after PBSC-T ($n = 18$) | 9 | 8–10 |
| Day platelet $\geqslant 20 \times 10^9 l^{-1}$ | 12 | 7–28 |
| No. of RBC units transfused | 4 | 2–8 |
| No. of platelet transfusions | 3.5 | 2–8 |
| $CD34^+$ cells ($\times 10^6 kg^{-1}$) | 10.2 | 0.7–25.1 |
| GM-CFUs ($\times 10^4 kg^{-1}$) | 109 | 9–419 |
| MNCs ($\times 10^8 kg^{-1}$) | 3.55 | 2.1–12.6 |

RBC, red blood cell; GM-CFU, colony-forming unit of granulocytes and macrophages; MNC, mononuclear cell; G-CSF, granulocyte colony-stimulating factor; PBSC-T, peripheral blood stem cell transplantation.

**Figure 2** Size of stem cell harvest ($n = 29$).

cessful, repeated leucocytopheresis procedures failed to harvest haemopoietic progenitor cells. Microscopic examination of a bone marrow specimen showed hypocellularity in the absence of myelodysplastic features. Cytogenetic analysis was unremarkable. The first four patients underwent PBSC-T combined with autologous bone marrow reinfusion, while the other 24 patients received PBSC-T alone.

*Bone marrow recovery*

The main toxicity of CTC chemotherapy consisted in bone marrow suppression (Table II). All patients had periods of absolute neutropenia and required platelet and red blood cell transfusions. In the 18 patients who received G-CSF after PBSC-T, the granulocyte counts had recovered to at least $500 \times 10^6 l^{-1}$ within a median of 9 days (range 8-10) *vs* a median of 16 days (range 11-28) in the ten patients who did not receive G-CSF following reinfusion ($P = <0.001$) (Table II, Figure 3). The number, median and range of progenitor cells reinfused, as reflected in the number of $CD34^+$ cells, GM-CFUs and MNCs, was nearly identical in patients who did or did not receive G-CSF after PBSC-T (data not shown). The delayed granulocyte recovery observed in the patients without G-CSF post transplant confirms recently published data (Spitzer *et al.*, 1994).

Platelet transfusion independence was achieved within a median of 10 days (range 7-16) in the patients who received G-CSF after PBSC-T. Platelet recovery did not significantly differ between patients who received or did not receive G-CSF: a median of 12 days (range 7-28) was required (Table II).

*Infectious complications*

During the neutropenic phase following autotransplantation, 27 patients (96%) developed fever > 38°C, and in 14 of these the origin of the fever could be identified. In four patients, the fever was accompanied by positive blood cultures for *S taphylococcus epidermidis*; in two patients blood cultures revealed *S. aureus*, and in another one a *Bacillus* species was cultured from the blood (see below). In one patient, an adenovirus was cultured from a bronchial washing. In four patients, chest radiographs suggested pulmonary infiltration; in only one of these was a positive culture obtained from the sputum. Herpes zoster was encountered once. In one patient, fever was thought to originate from multiple pulmonary emboli which occurred in the absence of apparent thrombosis or cardiac valve abnormalities. Positive blood cultures of Gram-negative or fungal organisms were not observed.

Thirteen patients (46%) developed fever without an indication of an infectious origin. They were empirically treated with vancomycin, 500 mg q.i.d, and ceftazidime, 2 g three times daily until granulocyte recovery ($\geqslant 500 \times 10^6$ $l^{-1}$).

Despite the significantly faster granulocyte recovery in patients who received G-CSF after PBSC-T, the percentage of patients developing temperature >38°C and the duration of fever did not differ when compared with patients who did not receive G-CSF after reinfusion. However, the duration of hospitalisation was significantly shortened by the use of G-CSF post transplant: 14 days (median; range 10-20) in patients who received G-CSF compared with 17 days (median; range 13-26) in those who did not ($P = <0.001$) (data not shown).

**Figure 3** Time to granulocyte recovery ($500 \times 10^6 l^{-1}$), with (solid line) or without (dotted line) the use of G-CSF after PBSC-T.

*Organ toxicity*

During CTC chemotherapy, all patients experienced nausea and vomiting for a median period of 11 days (range 5-28) (Table III), resulting in a median weight loss of 3 kg (range + 2.5 to − 9 kg). Three patients received total parenteral nutrition, starting after PBSC-T, because of a >5% loss of pretransplant weight.

Mucositis was usually mild; WHO grade 3 was observed in only 21% of patients. Twenty patients (71%) developed a skin rash which generally coincided with the empiric administration of broad-spectrum antibiotics and which resolved with their discontinuation (Table III).

Cardiac failure associated with the administration of high-dose cyclophosphamide was not observed. One patient developed multiple pulmonary emboli for which she received conventional treatment with heparin and coumarin derivatives. None of the patients complained of tinnitus or hearing loss, but audiograms were not routinely obtained. Peripheral neuropathy was not encountered.

Biochemical analysis showed brief and reversible elevations of liver function tests, mainly transaminases and gamma-glutamyltransferase (γ-GT) with maximum values of twice the upper limit of normal, usually peaking on day 0 (transaminases) and day 9 (γ-GT). Renal function was undisturbed.

*Catheter-related complications*

In 18 patients (64%), Hickman-catheter related complications were observed (Table IV). In 13 patients (46%), cultures of blood drawn from the catheter yielded Gram-positive organisms, occurring in five patients within 48 h of catheter implantation. Infection at the entry site or along the subcutaneous tunnel of the catheter was not observed. No catheter had to be removed because of infection.

Catheter-induced major vein thrombosis, requiring removal of the Hickman, occurred in five patients (18%) (Table IV). In two patients, thrombosis developed 24 h and 1 week after implantation of the Hickman catheter. In these patients, peripheral stem cells were successfully harvested using a similar catheter inserted in the femoral vein; high-dose chemotherapy was later administered through a small double-lumen catheter, not subcutaneously tunnelled, using the unaffected contralateral subclavian vein. In one of these

**Table III** Non-haematological toxicity ($n = 28$)

| | n | *WHO grade median (range)* | *duration (days) median (range)* | *start (day)[a] median (range)* |
|---|---|---|---|---|
| Nausea/vomiting | 28 | 3 (2-4) | 11 (5-28) | −4 (−6−−2) |
| Diarrhoea | 26 | 3 (2-3) | 8 (3-19) | 2 (−5-10) |
| Mucositis | 28 | 2 (1-3) | 5 (3-13) | 3 (1-10) |
| Rash | 20 | 2 (1-2) | 7 (1-11) | 8 (0-11) |

[a]PBSC-T on day 0. PBSC-T, peripheral blood stem cell transplantation.

**Table IV** Catheter-related complications

| | No. of patients | No. of events |
|---|---|---|
| Infection[a] | 13 | 17 |
| Major vein thrombosis | 5 | 6 |
| Flow obstruction requiring streptokinase (see text) | 10 | 10 |
| Untimely removal | 5 | 6 |

[a]Defined as positive culture of blood withdrawn from the catheter.

patients, a recurrent thrombotic complication required removal of the second catheter as well, and a third catheter was inserted in the femoral vein. In the other three patients, thrombosis required removal of the catheter 2 and 3 weeks before and on day 15 after PBSC-T.

In ten patients (36%), backflow obstruction of the catheter occurred, leading to the inability to withdraw blood from at least one of the catheter lumina. Patency could be regained in all cases by a 12–24 h infusion of streptokinase, in a dose of 1000 U per lumen per hour (Table IV). This low-dose fibrinolytic therapy was effective and uncomplicated despite platelet transfusion dependence in one of these patients.

The only reason to remove Hickman catheters before the end of treatment was symptomatic major vein thrombosis (five cases) (Table IV). Fracture of the Hickman catheter or spontaneous migration requiring removal did not occur.

### Discussion

In 29 young and chemotherapy-naive patients with high-risk breast cancer, peripheral blood progenitor cells were mobilised using G-CSF and moderate doses of chemotherapy, and 28 of these subsequently underwent high-dose chemotherapy followed by peripheral stem cell transplantation. Stem cell mobilisation was successful in all but one of the patients and typically required only two or three leucocytopheresis sessions. The high-dose regimen was reasonably well tolerated, and life-threatening toxicity did not occur. All transplanted patients had rapid engraftment with a median time to neutrophil recovery (neutrophils > $0.5 \times 10^9 l^{-1}$) of 9 days in patients who received G-CSF following reinfusion (Table II, Figure 3) and a median time to platelet transfusion independence of 10 days. Despite the carboplatin dose in this CTC regimen, which is twice as high as that employed in the STAMP V regimen of the Boston group (Antman *et al.*, 1992), no renal function impairment was observed and no symptomatic hearing loss or neuropathy was reported by the patients. Hepatic toxicity, which precluded further carboplatin dose escalation in the Boston phase I study of CTCb (Eder *et al.*, 1990), was limited to minor elevations of alanine (ALAT) and aspartate aminotransferases (ASAT), which had usually resolved by the second day after stem cell reinfusion. In addition, no congestive heart failure associated with high-dose cyclophosphamide was observed in this patient group.

In our experience, CTC is a high-dose regimen with little or no severe extramedullary toxicity. We have shown that the same regimen can be administered twice in a tandem transplantation strategy for the salvage treatment of germ cell cancer (Rodenhuis *et al.*, 1994). Even then, organ toxicity is relatively minor, although high-frequency hearing loss and increase in severity of pre-existent cisplatin neuropathy were common in these heavily pretreated patients. The conclusion that CTC is a safe regimen for adjuvant chemotherapy studies in breast cancer appears to be justified.

Although life-threatening toxicity of CTC was absent and the time to haemopoietic reconstitution was brief (particularly when G-CSF was used after stem cell reinfusion), the reversible non-life threatening toxicity was considerable. Nausea, vomiting and diarrhoea were substantial and occurred in almost all patients despite high doses of antiemetics and antidiarrhoeal agents (Table III). Neutropenic fevers were common, occurring in all but one patient. Skin rashes of uncertain origin were observed in 71% of the patients, some of which may have resulted from allergies to antimicrobial agents and some of which may have represented skin toxicity of thiotepa (Wolff *et al.*, 1990). Recent reports in the news media, suggesting that high-dose chemotherapy for breast cancer could be administered in an out-patient setting with patients reporting daily to the transplantation clinic, clearly apply to either less toxic chemotherapy regimens or to a small subset of patients. Most of our patients required hospitalisation from the start of chemotherapy until approximately 2 weeks after the stem cell reinfusion.

The stem cell mobilisation strategy employed was convenient and efficient. The standard chemotherapy designed for efficacy in breast cancer could very well serve as a mobilising regimen and the low dose of G-CSF (300 μg total dose irrespective of body weight, as opposed to 10 $\mu g\ kg^{-1}$ as recommended by most authors) led to high peripheral $CD34^+$ cell counts that allowed the harvest of sufficient numbers of stem cells in three or fewer leucocytopheresis sessions in 26 of 29 patients (Figure 2). In only one patient was no mobilisation at all observed. No cause of this failure could be identified: the bone marrow was hypocellular but showed normal morphology, and cytogenetic abnormalities were absent, arguing against the possibility of a myelodysplastic syndrome. The patient was taken off study and an autologous bone marrow transplantation was not attempted.

The clinically most important problem of the peripheral stem cell harvests consisted of complications related to the indwelling intravenous catheter. Sixty four per cent of all patients presented with catheter problems on one or more occasions during the course of their adjuvant treatment. The most serious problem was major vein thrombosis, which almost invariably appeared to originate from the subclavian vein, into which the large-bore silicone Hickman catheter had been inserted. We have previously reported significant haemorrhagic complications when attempting to dissolve the thrombosis employing systemic lose-dose fibrinolysis with recombinant tissue plasminogen activator while leaving central venous catheters *in situ* (Rodenhuis *et al.*, 1993). As a result, it was our policy to promptly remove the catheter and to start the patients on intravenous heparin.

The relatively high frequency of catheter-associated venous thrombosis may be the result of the large-bore catheters that are being used to facilitate the high blood flow required for efficient leucocytopheresis sessions (Haire *et al.*, 1990). Since the large majority of the patients required only 2–3 days of aphereses and four of five thrombotic events occurred one to several weeks after the insertion of the catheter, it may be prudent routinely to insert the catheter immediately before the first stem cell harvest, and to remove it immediately after completion of harvesting. A second but smaller and possibly less thrombogenic central venous catheter could be inserted later, preferably just before the start of high-dose chemotherapy.

High-dose chemotherapy with peripheral blood progenitor cell transplantation is clearly developing into a practical and safe modality in the adjuvant treatment of breast cancer. It continues, however, to be associated with substantial reversible toxicity, requiring prolonged hospitalisation and intensive supportive care. It can only be employed at considerable cost, in terms of both loss of well-being of the patient and her family and cost to society. Whether or not these costs are justified depends on the presence of a survival benefit which can only be studied through prospective randomised trials, some of which are now in progress in Europe and in the USA.

**Acknowledgements**
This study was supported in part by a grant from the Schumacher–Kramer Foundation.

**References**

ANTMAN KH. (1992). Dose-intensive therapy in breast cancer. In *High-dose Cancer Therapy*, (eds) Armitage JO, Antman KH pp.701–708. Williams & Wilkins: Baltimore.

ANTMAN KH, AYASH L, ELIAS A, WHEELER C, HUNT M, EDER JP, TEICHER BA, CRITCHLOW J, BIBBO J, SCHNIPPER LE AND FREI III E. (1992). A phase II study of high-dose cyclophosphamide, thiotepa and carboplatin with autologous marrow support in women with measurable advanced breast cancer responding to standard-dose therapy. *J. Clin. Oncol.*, **10**, 102–110.

BRONCHUD MH, HOWELL A, CROWTHER D, HOPWOOD P, SOUZA L AND DEXTER TM. (1989). The use of granulocyte colony-stimulating factor to increase intensity of treatment with doxorubicin in patients with advanced breast and ovarian cancer. *Br. J. Cancer*, **60**, 121–125.

DE VRIES EGE, RODENHUIS S, SCHOUTEN HC, HUPPERETS PSGJ, BLIJHAM GH, BONTEBAL M, RODENBURG CJ AND MULDER NH. (1994). Phase II study of intensive chemotherapy with autologous bone marrow transplantation in patients in complete remission of disseminated breast cancer (abstract). *Proc. Am. Soc. Clin. Oncol.*, **13**, 87.

EARLY BREAST CANCER TRIALISTS' COLLABORATIVE GROUP. (1992). Systemic treatment of early breast cancer by hormonal, cytotoxic or immune therapy. *Lancet*, **339**, 1–15, 71–85.

EDER JP, ELIAS A, SHEA TC, SCHRYBER SM, TEICHER BA, HUNT M, BURKE J, SIEGEL R, SCHNIPPER LE, FREI III E AND ANTMAN K. (1990). A phase I–II study of cyclophosphamide, thiotepa and carboplatin with autologous bone marrow transplantation in solid tumor patients. *J. Clin. Oncol.*, **8**, 1239–1245.

FISHER B, WICKERHAM DLC AND REDMOND CK. (1992). Recent developments in the use of systemic adjuvant therapy for the treatment of breast cancer. *Semin. Oncol.*, **19**, 263–277.

GIANNI AM, BREGNI M, SIENA S, ORAZI A, STERN AC, GANDOLA L AND BONADONNA G. (1990). Recombinant human granulocyte–macrophage colony-stimulating factor reduces hematologic toxicity and widens clinical applicability of high-dose cyclophosphamide treatment in breast cancer and non-Hodgkin's lymphoma. *J. Clin. Oncol.*, **8**, 768–778.

GIANNI AM, SIENA A, BREGNI M, DI NICOLA M, OREFICE S, LUINI A, GRECO M, ZUCALI R, VALAGUSSA P AND BONADONNA G. (1992). Growth factor-supported high-dose sequential (HDS) adjuvant chemotherapy in breast cancer with $\geqslant$ 10 positive nodes (abstract 68). *Proc. Am. Soc. Clin. Oncol.*, **11**, 60.

HAIRE WD, LIEBERMAN RP, EDNEY J, VAUGHAN WP, KESSINGER A, ARMITAGE JO AND GOLDSMITH JC. (1990). Hickman catheter-induced thoracic vein thrombosis. Frequency and long-term sequelae in patients receiving high-dose chemotherapy and marrow transplantation. *Cancer*, **66**, 900–908.

KLUMPP TE, GOLDBERG SL, MANGAN KF AND MACDONALD JS. (1994). High dose cyclophosphamide, etoposide, and carboplatin (CEC) with autologous stem cell rescue for chemosensitive metastatic and high risk breast cancer (abstract). *Proc. Am. Soc. Clin. Oncol.*, **13**, 111.

LIVINGSTON RB. (1994). High-dose consolidation for stage IV breast cancer. In *American Society of Clinical Oncology, Educational Book*, (eds) Gastineau DA, Ramsay NK. pp. 74–79. Bostrom Corporation: Dallas.

MULDER NH, SLEIJFER DTH, DE VRIES EGE, MULDER POM VD, GRAAF WTA, VD PLOEG, E DOLSMA WV AND WILLEMSE PHB. (1993). Intensive chemotherapy with autologous bone marrow reinfusion in patients with breast cancer and more than 5 involved lymph nodes (abstract). *Proc. Am. Soc. Clin. Oncol.*, **12**, 104.

OLIVOTTO JA, BAJDIK CD, MATH M, PLENDERLEITH JH, COPPIN CM, GELMON KA, JACKSON SM, RAGAZ J, WILSON KS AND WORTH A. (1994). Adjuvant systemic therapy and survival after breast cancer. *N. Engl. J. Med.*, **330**, 805–810.

PETERS WP, ROSS M, VREDENBURGH JJ, MEISENBERG B, MARKS LB, WINER E, KURTZBERG J, BAST RC, JONES R, SHPALL E, WU K, ROSNER G, GILBERT C, MATHIAS B, CONIGLIO D, PETROS W, HENDERSON IC, NORTON L, WEISS RB, BUDMAN D AND HURD D. (1993). High-dose chemotherapy and autologous bone marrow support as consolidation after standard-dose adjuvant therapy for high-risk primary breast cancer. *J. Clin. Oncol.*, **11**, 1132–1143.

RODENHUIS S, BAARS JW, SCHORNAGEL JH, VLASVELD LT, MANDJES I, PINEDO HM AND RICHEL DJ. (1992). Feasibility and toxicity study of a high-dose chemotherapy regimen for autotransplantation incorporating carboplatin, cyclophosphamide and thiotepa. *Ann. Oncol.*, **3**, 855–860.

RODENHUIS S, VAN T HEK LGFM, VLASVELD LT, KROGER R, DUBBELMAN R AND VAN TOL RGL. (1993). Thrombotic major vein obstruction as a complication of a central venous catheter: thrombolytic therapy with recombinant tissue plasminogen activator. *Thorax*, **48**, 558–559.

RODENHUIS S, VAN DER WALL E, TEN BOKKEL HUININK WW, SCHORNAGEL JH, RICHEL DJ AND VLASVELD LT. (1994). Pilot study of a high-dose carboplatin-based salvage strategy for relapsing or refractory germ cell cancer. *Cancer Invest.* (in press).

SPITZER G, ADKINS DR, SPENCER V, DUNPHY FR, PETRUSKA PJ, VELASQUEZ WS, BOWERS CE, KRONMUELLER N, NIEMEYER R AND MCINTYRE W. (1994). Randomized study of growth factors post-peripheral-blood stem-cell transplant: neutrophil recovery is improved with modest clinical benefit. *J. Clin. Oncol.*, **12**, 661–670.

VAN DER WALL E, RICHEL DJ, KUSUMANTO YH, RUTGERS EJT, SCHORNAGEL JH, SCHAAKE-KONING CCE, PETERSE JL AND RODENHUIS S. (1993). Feasibility study of FEC-chemotherapy with dose-intensive epirubicin as initial treatment in high-risk breast cancer. *Ann. Oncol.*, **4**, 791–792.

VAN DER WALL E, RICHEL DJ, HOLTKAMP MJ, SLAPER-CORTENBACH ICM, VAN DER SCHOOT CE AND DALESIO O. (1994). Bone marrow reconstitution after high-dose chemotherapy and autologous peripheral blood progenitor cell transplantation: effect of graft size. *Ann. Oncol.* (in press).

WOLFF SN, HERZIG RH, FAY JW, LE MAISTRE CF, BROWN RA, FREI-LAHR D, STRANJORD S, GIANNONE L, COCCIA P, WEICK JL, ROTHMAN SA, KRUPP KR, LOWDER J, BOLWELL B AND HERZIG GP. (1990). High-dose N, N', N''-triethylenethiophosphoramide (thiotepa) with autologous bone marrow transplantation: phase I studies (review). *Semin. Oncol.*, **17**, (Suppl. 3) 2–6.

WOOD WC, BUDMAN DR, KORZUN AH, COOPER MR, YOUNGER J, HART RD, MOORE A, ELLERTON JA, NORTON L, FERREE CR, COLANGELO BALLOW A, FREI III E AND HENDERSON IC. (1994). Dose and dose intensity of adjuvant chemotherapy for stage II, node-positive breast carcinoma. *N. Engl. J. Med.*, **330**, 1253–1259.